# Variable clinical expression of a Belgian *TGFB3* founder variant suggests the presence of a genetic modifier

**DOI:** 10.3389/fgene.2023.1251675

**Published:** 2023-08-31

**Authors:** Melanie H. A. M. Perik, Emmanuela Govaerts, Steven Laga, Inge Goovaerts, Johan Saenen, Emeline Van Craenenbroeck, Josephina A. N. Meester, Ilse Luyckx, Inez Rodrigus, Aline Verstraeten, Lut Van Laer, Bart L. Loeys

**Affiliations:** ^1^ Cardiogenomics and Functional Genomics, Center for Medical Genetics, University of Antwerp and Antwerp University Hospital, Antwerp, Belgium; ^2^ Department of Cardiology, AZ Dimpna, Geel, Belgium; ^3^ Department of Cardiac Surgery, Antwerp University Hospital, Antwerp, Belgium; ^4^ Department of Cardiology, Antwerp University Hospital, Antwerp, Belgium; ^5^ Department of Human Genetics, Radboud University Medical Center, Nijmegen, Netherlands

**Keywords:** Loeys–Dietz syndrome (LDS), *TGFB3*, thoracic aortic aneurysm and dissection (TAAD), founder, genetic modifiers, connective tissue disorder, variable expressivity

## Abstract

**Background:** *TGFB3* variants cause Loeys–Dietz syndrome type 5, a syndromic form of thoracic aortic aneurysm and dissection. The exact disease phenotype is hard to delineate because of few identified cases and highly variable clinical representation.

**Methodology:** We provide the results of a haplotype analysis and a medical record review of clinical features of 27 individuals from 5 different families, originating from the Campine region in Flanders, carrying the NM_003239.5(*TGFB3*):c.787G>C p.(Asp263His) likely pathogenic variant, dbSNP:rs796051886, ClinVar:203492. The Asp^263^ residue is essential for integrin binding to the Arg-Gly-Asp (RGD) motif of the TGFβ3-cytokine.

**Results:** The haplotype analysis revealed a shared haplotype of minimum 1.92 Mb and maximum 4.14 Mb, suggesting a common founder originating >400 years ago. Variable clinical features included connective tissue manifestations, non-aneurysmal cardiovascular problems such as hypertrophic cardiomyopathy, bicuspid aortic valve, mitral valve disease, and septal defects. Remarkably, only in 4 out of the 27 variant-harboring individuals, significant aortic involvement was observed. In one family, a 31-year-old male presented with type A dissection. In another family, the male proband (65 years) underwent a Bentall procedure because of bicuspid aortic valve insufficiency combined with sinus of Valsalva of 50 mm, while an 80-year-old male relative had an aortic diameter of 43 mm. In a third family, the father of the proband (75 years) presented with ascending aortic aneurysm (44 mm).

**Conclusion:** The low penetrance (15%) of aortic aneurysm/dissection suggests that haploinsufficiency alone by the *TGFB3* variant may not result in aneurysm development but that additional factors are required to provoke the aneurysm phenotype.

## 1 Introduction

Loeys–Dietz syndrome (LDS) is an autosomal dominant connective tissue disorder presenting with cardiovascular, skeletal, and craniofacial features. It has significant clinical overlap with other connective tissue disorders such as Marfan syndrome [MIM: 154700]. Overlapping features include thoracic aortic aneurysm and dissection (TAAD), pectus deformity, scoliosis, joint laxity, arachnodactyly, and talipes equinovarus. Discriminative features include bifid uvula, cleft palate, and arterial tortuosity. Additionally, LDS patients are commonly more severely affected, with dissections and ruptures occurring at smaller aortic diameters, younger ages, and throughout the arterial tree (Meester et al., 2017). Six LDS genes have been identified to date, i.e., *TGFBR1* (Loeys et al., 2005), *TGFBR2* (Loeys et al., 2005), *SMAD2* (Micha et al., 2015), *SMAD3* (van de Laar et al., 2011), *TGFB2* (Lindsay et al., 2012), and *TGFB3* (Bertoli-Avella et al., 2015) [MIM: 190181, 190182, 601366, 603109, 190220, 190230], which encode cytokines, receptors, or intracellular effectors of the transforming growth factor (TGFß) pathway. LDS variants exert a loss-of-function effect, ultimately resulting in a paradoxical upregulation of the TGFβ pathway at the tissue level. Between LDS patients, disease severity can differ depending on the underlying gene defect. When compared to the other subtypes, *TGFB3*-related LDS (LDS5; [MIM: 615582]) is at the milder end of the LDS spectrum and presents with fewer non-vascular features and a later TAA(D) onset (Bertoli-Avella et al., 2015). Hitherto, 26 pathogenic/likely pathogenic *TGFB3* variants^1^ have been published in 94 different individuals of which 46% are missense, 7% frameshift, 21% nonsense, and 25% splice-altering variants (Supplementary Table S1) (Schepers et al., 2018; Hussein et al., 2021). Using routine TAA(D) panel genetic testing, we identified five LDS-like families from the Campine region segregating a NM_003239.5(*TGFB3*):c.787G>C p.(Asp263His) missense variant, dbSNP: rs796051886, ClinVar: 203492. Functional work has shown that Asp263 is part of the Arg-Gly-Asp (also known as RGD) motif which is key for the interaction with integrins. Abolishing the RGD motif at the cDNA level (RGE) (Munger et al., 1998) or protein level (AGD, RAD, RGA) (Ludbrook et al., 2003) demonstrated blockage of integrin interaction. As such, abolishing this highly conserved RGD sequence disturbs the function of TGFβ3. In this report, we demonstrate the existence of a common ancestor and describe a remarkably low penetrance of TAA(D) in these five families.

## 2 Materials and methods

### 2.1 Haplotyping

The presence of a shared genomic region flanking both sides of the p.(Asp263His) variant between the 5 families under investigation was evaluated using 11 highly polymorphic short tandem dinucleotide repeat markers (D14S1065, D14S258, D14S1028, D14S1047, D14S61, D14S270, D14S983, D14S74, D14S287, D14S1037, and D14S1044), which were selected using the Map Viewer (NCBI, Build GRCh37). Marker heterozygosity of the 11 short tandem dinucleotide repeat markers ranged from 0.63 to 0.83, with a mean of 0.75 (Marshfield comprehensive human genetic maps). Forward primers contained an M13-tail at the 5′ end and were labeled with 6-carboxyfluorescein. PCR products were run on an ABI Prism 3130xl Genetic Analyzer (Applied Biosystems) with GeneScan 500 ROX as an internal sizing standard. By using the ABI GeneMapper Software v3.7 (Applied Biosystems), amplicon sizes were determined and compared between a total of six individuals of the five different families carrying the p.(Asp263His) variant. Subsequently, an age estimation of the most recent common ancestor was made using the formula given by Marroni et al. (2008) [ $n=\frac{\log\left(2/5\right)}{\log\left(1-0.04\right)}$ ], for the probability of observing a recombination event between two markers, i.e., the distance between these markers in centimorgan (cM).

### 2.2 Clinical and molecular diagnosis

In the Cardiogenetics Clinic of the Antwerp University Hospital, peripheral blood was collected in ethylenediaminetetraacetic acid (EDTA) tubes, and genomic DNA was extracted using the Maxwell^®^ RSC DNA extraction system with the Maxwell^®^ RSC Cultured Cells DNA Kit (Promega), following the manufacturer’s protocol. Genetic testing for Marfan and LDS-like patients was done using a next-generation sequencing–based assay comprising 34 TAA(D)-related genes (Supplementary Table S2) as previously described (Proost et al., 2015). In short, a custom HaloPlex target enrichment kit (Agilent Technologies) was used with probes designed with the HaloPlex design wizard (Agilent Technologies), following the supplier's protocol (version F1, July 2015). Next-generation sequencing was then performed using 150-bp paired-end sequencing reads on a MiSeq (Illumina). Adapter sequences were trimmed using cutadapt v1.2.1, and reads were trimmed by an in-house developed tool when the Phred scores exceeded 30 and mapped using BWA MEM v.0.7.4 and GATK toolkit v2.8.1, and then genotyped using the GATK UnifiedGenotyper. Sensitivity was increased by combining multisample and single-sample genotyping, and by using the GATK LeftAlignAndTrim method, the notation of insertions and deletions were normalized. Next, the samples were filtered using VariantDB (Vandeweyer et al., 2014) based on the frequency using the dbSNP (NCBI), ExAC (v.02), and 1000 Genomes project, and a combination of prediction programs (PolyPhen MutationTaster, SIFT, and CADD score) was used for variant classification. An integrative genomic viewer (IGV) was used for visual inspection of interesting variants, and by using Alamut^®^, the splice site effects were evaluated.

Sanger sequencing was used to confirm the variant(s) of interest and check segregation in relatives. Polymerase chain reaction (PCR) products were obtained by Touchdown PCR on a thermal cycler with M13-containing primers (IDT, for the p.Asp263His variant forward: GTT​TTC​CCA​GTC​ACG​ACA​CAC​CTC​CCT​CGC​AGA​CT, reverse: CAG​GAA​ACA​GCT​ATG​ACT​TGA​GTG​TGG​CTT​GGC​TCT​G. By using the BigDye Terminator Cycle Sequencing Kit (Applied Biosystems) and capillary electrophoresis on an ABI PRISM 3130xl Genetic Analyzer system (Applied Biosystems), the samples of interest were sequenced. The sequences were then analyzed with CLC DNA workbench (v.5.7.1; CLC Bio, Denmark). For each Marfan syndrome and LDS-like proband and his/her relatives, a medical history was obtained, and a physical examination was performed with specific focus on connective tissue and cardiovascular manifestations. Patient's aortic size was measured with transthoracic echocardiography, CT angiography, or MR angiography. The Z-scores were calculated based on Devereux et al. (2012).

## 3 Results

### 3.1 *TGFB3* founder variant

**FIGURE 1:**
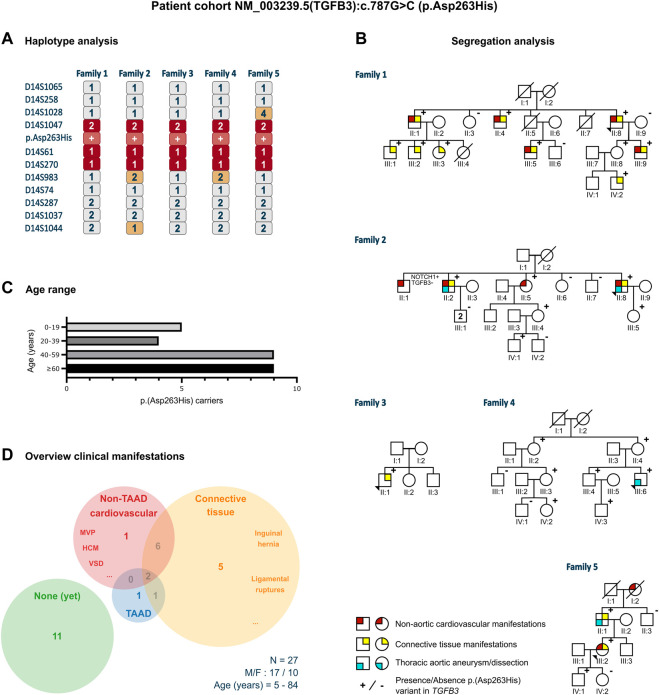
Clinical characteristics of the p.(Asp263His) patient cohort. **(A)** Proband of all five families that underwent haplotype analysis for 11 markers flanking the p.(Asp263His) likely pathogenic variant showing a shared minimal region between marker D14S1047 and D14S270 and a maximal region between marker D14S1028 and D14S983. **(B)** Pedigrees with clinical presentation of the families harboring the p.(Asp263His) variant with regard to non-aortic cardiovascular manifestations, thoracic aortic aneurysm or dissection, and connective tissue features. **(C)** Age distribution of the p.(Asp263His) carriers over the five families. **(D)** Distribution of clinical manifestations among the family members of the p.(Asp263His) cohort. F, female; HCM, hypertrophic cardiomyopathy; M, male; MVP, mitral valve prolapse; N, number of patients; TAAD, thoracic aortic aneurysm; and VSD, ventricular septal defect.

Twenty-seven individuals from five different families carried an identical, heterozygous variant in *TGFB3*, p.(Asp263His) (c.787G>C). This variant was absent from gnomAD and 1000 Genomes Project (PM2) but present in ClinVar (PP5). The phyloP (score: 5.605) and phastCons (score: 1) predicted conservation for this amino acid (PP3). The functional evidence showed that the abolishment of the RGD motif at the cDNA level (RGE) of TGFβ1 (Munger et al., 1998) and at the protein level (AGD, RAD, RGA) of both TGFβ1 and TGFβ3 (Ludbrook et al., 2003) blocks integrin interaction (PS3). The phenotype of the families was consistent with other LDS types and more specifically with other (likely) pathogenic *TGFB3* variants that included aortic aneurysm, bifid uvula, arachnodactyly, scoliosis, and palate deformities (PP4). Therefore, based on the ACMG criteria, this variant was classified as likely pathogenic. The haplotype analysis, using 11 polymorphic short tandem repeat markers flanking the variant in the probands of the families, revealed a shared haplotype of minimum 1.92 Mb (D14S1047-D14S270, 2.13 cM) and maximum 4.14 Mb (D14S1028-D14S983, 6.54 cM), thus confirming a founder effect (Figure 1A). The most recent common ancestor was estimated to live 22 generations ago, corresponding to approximately 434 years.

### 3.2 Clinical manifestations

#### 3.2.1 Family 1

The 64-year-old male proband (II:8) was initially referred because of hypertrophic cardiomyopathy. Cardiogenetic testing did not reveal a pathogenic variant in known hypertrophic cardiomyopathy genes but identified the *TGFB3* p.(Asp263His) likely pathogenic variant. The aortic measurements were normal. The family history revealed that a non-carrying brother (II:7) had died of an abdominal aortic dissection at 64 years. He had a history of heavy smoking, hypercholesterolemia, and hypertension. A daughter (III:4) of another brother (II:1) of the proband died suddenly at 4 years, but no autopsy was performed. A third brother (II:5) succumbed to mesothelioma at 59 years. Subsequent segregation analysis revealed nine additional p.(Asp263His)-harboring individuals (two females and seven males) with an age range of 7–84 years. None of them showed significant aortic dilatation, with the largest aortic root being Z = 2.0 in a 36-year-old male (III:9). The non-aortic (cardio)vascular features in variant carriers included tricuspid aortic valve insufficiency, mitral valve insufficiency (*n* = 2), ventricular septal defect (surgery when aged 1 year), concentric and septal hypertrophy (IVSd 14 mm in III:5, aged 49 years, and IVSd 16 mm in II:4, aged 77 years), and coronary disease. The connective tissue features included hernia inguinalis, pes planus (*n* = 2), myopia (*n* = 2), soft skin, Peyronie's disease, Dupuytren's disease (*n* = 2), gonarthrosis, vertebral disk hernia (*n* = 2), easy bruising, and torn ligaments (ankle and shoulder).

#### 3.2.2 Family 2

The proband, a 74-year-old male (II:8), underwent a Bentall procedure (65 years) because of insufficiency of a bicuspid aortic valve (BAV) along with a sinus of Valsalva aortic aneurysm of 50 mm (Z = 5.2). Other cardiovascular and connective tissue features included mitral valve insufficiency and varices. Familial segregation identified p.(Asp263His) in two of the five siblings. The proband’s 55-year-old variant-harboring sister (II:5) had normal aortic measurements but suffered from atrial fibrillation at 53 years. Her variant-positive daughter (III:4, 44 years) did not present with TAA(D). The proband’s variant-positive older brother (II:2, 80 years) presented with an aortic root diameter of 43 mm (Z = 2.1), concentric hypertrophy, disk herniation, and carried a pacemaker because of an AV block. His two sons (III:1) did not harbor the variant. The proband’s variant-positive daughter (III:5) had an aortic sinus of 34 mm (Z = 1.7) at 41 years but declined further follow-up. One brother (II:1), without p.(Asp263His), had an aortic valve replacement because of aortic insufficiency (77 years), a pacemaker due to an atrial flutter and a coronary artery bypass graft, and showed an aortic root dilatation (45 mm; Z = 2.7). He passed away at 83 years due to COVID-19. Predisposing cardiovascular risk factors, such as smoking and diabetes, were absent. A TAAD-gene panel in this brother revealed the presence of a variant of unknown significance in *NOTCH1* (c.6910T>G; p.(Leu2304Val)), which was absent in all other relatives.

#### 3.2.3 Family 3

The proband (II:1), a 15-year-old boy, was referred because of a bifid uvula, tall stature, and dolichostenomelia (arm span to height ratio: 1.05 and upper segment/lower segment ratio: 0.79). He also presented with mild scoliosis, arachnodactyly with a positive wrist and thumb sign, skin striae on the lower back, and pes planus. The aortic sinus measured 30 mm (Z = 0.0). Unfortunately, the parents declined segregation analysis and clinical follow-up. The family history was negative for TAA(D).

#### 3.2.4 Family 4

The 31-year-old male proband (III:6) presented with a type A dissection. His medical history was significant for growth hormone substitution during puberty and mandibular advancement surgery (22 years). He had no history of hypertension, dislocations, fractures, or wound-healing problems. The aortic dissection presented with pain in the lower legs and feet, complicated by renal insufficiency. He successfully underwent a Bentall surgery. His physical examination revealed no skeletal overgrowth but long, narrow facies with mild retrognathia (post-surgery) and high palate with normal uvula. Furthermore, we observed low muscle mass and stiff joints but no pectus deformity, scoliosis, or striae. Segregation analysis revealed six additional p.(Asp263His)-harboring individuals (four females and two males, age range 5–76 years), but none showed TAAD or connective tissue findings.

#### 3.2.5 Family 5

A 50-year-old female (III:2) was referred for suspicion of long QT syndrome. No pathogenic variant in any of the known long QT syndrome genes was found, but she was coincidentally found to harbor the *TGFB3* p.(Asp263His) variant. Echocardiographic evaluation revealed normal aortic diameters. Her medical history was significant for inguinal hernia surgery. Segregation analysis demonstrated the presence of p.(Asp263His) in her father and son. Her father (II:1, 75 years) presented with an ascending aortic aneurysm (44 mm, Z = 6.4) and a history of early-onset varices and arthrosis. The son (IV:1) had normal aortic diameters at 25 years. A brother of the father (II:3) tested negative for the variant. The paternal grandmother of the proband (I:2) had an aortic valve replacement at 75 years and died at 88 years due to dementia.

### 3.3 Comparison with literature

**FIGURE 2:**
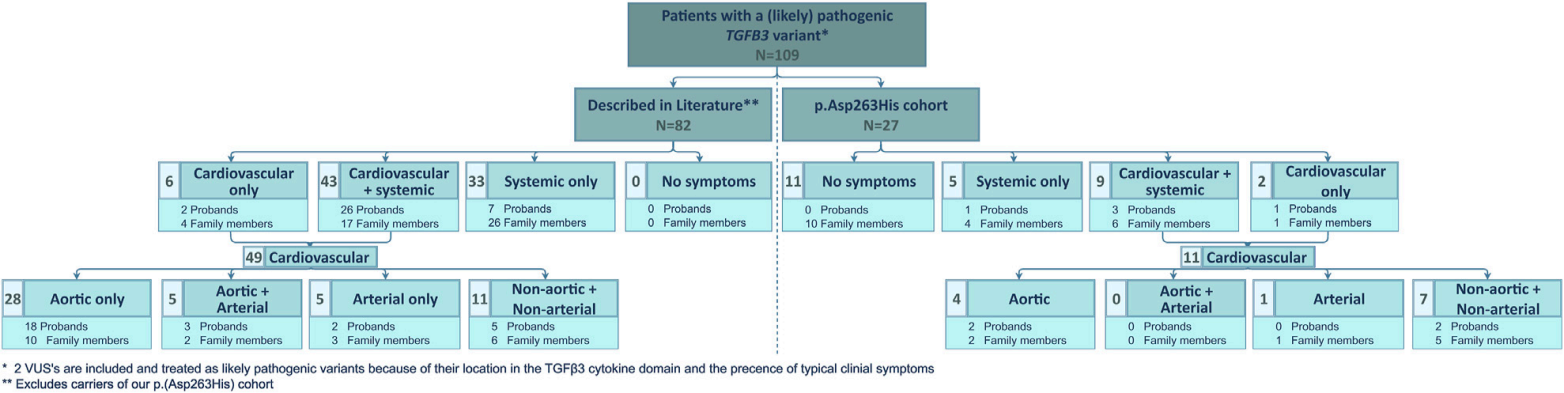
Overview of clinical manifestations of patients with a (likely) pathogenic *TGFB3* variant described in literature and comparison to the clinical manifestations present in our p.(Asp263His) cohort. N, number of patients and VUS, variant of uncertain significance.

We compared our low TAA(D) penetrance to that in other published LDS5 patients (Figure 2). From a literature search, we retained 94 published *TGFB3* probands and relatives (Supplementary Table S1) (Rienhoff et al., 2013; Matyas et al., 2014; Bertoli-Avella et al., 2015; Kuechler et al., 2015; Ziganshin et al., 2015; Overwater et al., 2018; Schepers et al., 2018; Marsili et al., 2020; Abdelhadi et al., 2021; Hussein et al., 2021). For our comparative analysis, 12 patients with the p.(Asp263His) variant were excluded from the literature cohort, as they were part of the families described in our cohort. Of the 82 remaining patients (35 probands, 47 relatives; M/F ratio: 46/36), only 33 (40%) presented with TAA(D). This is significantly higher than in our series (15%, 4/27), proportion test with Yates continuity correction *p* = 0.0288, but still shows significant non-penetrance for the aortic phenotype. Considering any arterial involvement, this number increases to 46% (38/82) in the literature and is significantly higher than in our cohort, where we reach 19% (5/27), *p* = 0.0194. The overall penetrance of the connective tissue findings in the literature cohort is as high as 93% (76/82), while in our cohort, only 52% (14/27) was affected. This is again significantly different between cohorts, *p* < 0.0001. These observations may be due to an underrepresentation of asymptomatic patients in publications.

## 4 Discussion

Five LDS-like families were found to harbor the identical likely pathogenic *TGFB3* p.(Asp263His) variant. In total, 27 variant-harboring individuals (10 females and 17 males; average age: 47 years, range: 5–84 years) were identified (Figures 1B, C). Only four presented with TAA(D) (average age: 63 years, range 31–81 years). Nine individuals displayed other cardiovascular findings (e.g., ventricular septal defects, valve insufficiencies, and variable conduction abnormalities; average age: 57 years, range: 10–84 years), and 14 had connective tissue findings (which included easy bruising, inguinal hernias, and ligament and tendon tears; average age: 55 years, range: 7–84 years). In total, 11 patients (average age: 38 years, range: 5–76 years) did not present any phenotypical abnormalities (Figure 1D).

The haplotype analysis revealed a shared haplotype of 1.92–4.14 Mb flanking the p.(Asp263His) variant, suggesting a common founder originating about 400 years ago. Although some family members presented multiple LDS-like connective tissue (*n* = 14) and non-TAA(D) cardiovascular (*n* = 9) features, the penetrance of aneurysmal disease in these families was low, with only 15% of variant-harboring individuals (4/27) presenting with TAA(D).

The low penetrance of TAA(D) and/or non-aortic arterial involvement in both our *TGFB3* p.(Asp263His) founder cohort and literature suggests the need for additional genetic or environmental modifiers to provoke the aneurysm phenotype. Although we did not find evidence that classic cardiovascular risk factors such as hypertension, smoking, diabetes, or hypercholesterolemia are the culprits in our families, we cannot rule them out. Of course, age could be an important factor. This is supported by the rather older age (average 63 years) by when the p.(Asp263His) variant–harboring individuals manifested aortic aneurysm/dissection, and this is corroborated by the literature data. From our literature review, the average age of “symptomatic” index patients (defined as TAA(D)) was 42 years (range 3–72 years), whereas it was 54 years (range 6–80 years) for the variant-harboring family members. Another potential explanation for the low penetrance would be the need for a second variant on the other *TGFB3* allele. This second event could be somatic (e.g., in aortic wall tissue), and also the expression of the modifying variants on the other alleles might contribute to increased penetrance. This is supported by the observation that “homozygous” *TGFB3* mutant patients presented with a more severe and early-onset phenotype (Marsili et al., 2020). Alternatively, second hits at other loci could also modulate the phenotypical expression. Finally, variability in the paradoxical upregulation of the TGFβ pathway (Bertoli-Avella et al., 2015) compensating for the haploinsufficiency of *TGFB3* expression might add to the phenotypic variation. Further research is necessary to shed a better light on the genetic and/or environmental modifying mechanisms.

## 5 Conclusion

From all LDS genes, *TGFB3* seems to have the lowest penetrance, both for vascular and connective tissue features. In our series, the overall aortic aneurysm penetrance was as low as 15%. The literature data confirmed a reduced penetrance of 40%. These observations suggest the need for a second environmental or heritable factor to elicit the aneurysmal phenotype of *TGFB3* (likely) pathogenic variant–harboring individuals.

## Data Availability

The data sets presented in this study can be found in online repositories. The names of the repository/repositories and accession number(s) can be found online at https://www.ncbi.nlm.nih.gov/, VCV000203492.2, and https://www.ncbi.nlm.nih.gov/snp/, rs796051886.

## Footnotes

1 For two variants of uncertain significance (VUS) located in the TGFβ3 cytokine domain, the clinical data contributed to the decision to classify these variants as likely pathogenic.

## References

[B1] Abdelhadi N. Zghouzi M. Sattar Y. Jokhadar M. Alraies M. C. (2021). Recurrent coronary artery fistulae and a novel transforming growth factor beta-3 mutation. Cureus 13 (9), e17780. 10.7759/cureus.17780 34659991 PMC8496650

[B2] Bertoli-Avella A. M. Gillis E. Morisaki H. Verhagen J. M. A. de Graaf B. M. van de Beek G. (2015). Mutations in a TGF-beta ligand, TGFB3, cause syndromic aortic aneurysms and dissections. J. Am. Coll. Cardiol. 65 (13), 1324–1336. 10.1016/j.jacc.2015.01.040 25835445 PMC4380321

[B3] Devereux R. B. de Simone G. Arnett D. K. Best L. G. Boerwinkle E. Howard B. V. (2012). Normal limits in relation to age, body size and gender of two-dimensional echocardiographic aortic root dimensions in persons ≥15 years of age. Am. J. Cardiol. 110 (8), 1189–1194. 10.1016/j.amjcard.2012.05.063 22770936 PMC3462295

[B4] Hussein D. Olsson C. Lagerstedt-Robinson K. Moreira T. (2021). Novel mutation of the TGF-beta 3 protein (Loeys-Dietz type 5) associated with aortic and carotid dissections: case report. Neurol. Genet. 7 (6), e625. 10.1212/NXG.0000000000000625 34549088 PMC8448523

[B5] Kuechler A. Altmuller J. Nurnberg P. Kotthoff S. Kubisch C. Borck G. (2015). Exome sequencing identifies a novel heterozygous TGFB3 mutation in a disorder overlapping with Marfan and Loeys-Dietz syndrome. Mol. Cell. Probes 29 (5), 330–334. 10.1016/j.mcp.2015.07.003 26184463

[B6] Lindsay M. E. Schepers D. Bolar N. A. Doyle J. J. Gallo E. Fert-Bober J. (2012). Loss-of-function mutations in TGFB2 cause a syndromic presentation of thoracic aortic aneurysm. Nat. Genet. 44 (8), 922–927. 10.1038/ng.2349 22772368 PMC3616632

[B7] Loeys B. L. Chen J. Neptune E. R. Judge D. P. Podowski M. Holm T. (2005). A syndrome of altered cardiovascular, craniofacial, neurocognitive and skeletal development caused by mutations in TGFBR1 or TGFBR2. Nat. Genet. 37 (3), 275–281. 10.1038/ng1511 15731757

[B8] Ludbrook S. B. Barry S. T. Delves C. J. Horgan C. M. (2003). The integrin alphavbeta3 is a receptor for the latency-associated peptides of transforming growth factors beta1 and beta3. Biochem. J. 369 (2), 311–318. 10.1042/BJ20020809 12358597 PMC1223078

[B9] Marroni F. Cipollini G. Peissel B. D’Andrea E. Pensabene M. Radice P. (2008). Reconstructing the genealogy of a BRCA1 founder mutation by phylogenetic analysis. Ann. Hum. Genet. 72, 310–318. 10.1111/j.1469-1809.2007.00420.x 18215206

[B10] Marsili L. Overwater E. Hanna N. Baujat G. Baars M. J. H. Boileau C. (2020). Phenotypic spectrum of TGFB3 disease-causing variants in a Dutch-French cohort and first report of a homozygous patient. Clin. Genet. 97 (5), 723–730. 10.1111/cge.13700 31898322

[B11] Matyas G. Naef P. Tollens M. Oexle K. (2014). De novo mutation of the latency-associated peptide domain of TGFB3 in a patient with overgrowth and Loeys-Dietz syndrome features. Am. J. Med. Genet. A 164A (8), 2141–2143. 10.1002/ajmg.a.36593 24798638

[B12] Meester J. A. N. Verstraeten A. Schepers D. Alaerts M. Van Laer L. Loeys B. L. (2017). Differences in manifestations of Marfan syndrome, Ehlers-Danlos syndrome, and Loeys-Dietz syndrome. Ann. Cardiothorac. Surg. 6 (6), 582–594. 10.21037/acs.2017.11.03 29270370 PMC5721110

[B13] Micha D. Guo D. C. Hilhorst-Hofstee Y. van Kooten F. Atmaja D. Overwater E. (2015). SMAD2 mutations are associated with arterial aneurysms and dissections. Hum. Mutat. 36 (12), 1145–1149. 10.1002/humu.22854 26247899

[B14] Munger J. S. Harpel J. G. Giancotti F. G. Rifkin D. B. (1998). Interactions between growth factors and integrins: latent forms of transforming growth factor-beta are ligands for the integrin alphavbeta1. Mol. Biol. Cell. 9 (9), 2627–2638. 10.1091/mbc.9.9.2627 9725916 PMC25536

[B15] Overwater E. Marsili L. Baars M. J. H. Baas A. F. van de Beek I. Dulfer E. (2018). Results of next-generation sequencing gene panel diagnostics including copy-number variation analysis in 810 patients suspected of heritable thoracic aortic disorders. Hum. Mutat. 39 (9), 1173–1192. 10.1002/humu.23565 29907982 PMC6175145

[B16] Proost D. Vandeweyer G. Meester J. A. Salemink S. Kempers M. Ingram C. (2015). Performant mutation identification using targeted next-generation sequencing of 14 thoracic aortic aneurysm genes. Hum. Mutat. 36 (8), 808–814. 10.1002/humu.22802 25907466

[B17] Rienhoff H. Y. Jr. Yeo C. Y. Morissette R. Khrebtukova I. Melnick J. Luo S. (2013). A mutation in TGFB3 associated with a syndrome of low muscle mass, growth retardation, distal arthrogryposis and clinical features overlapping with Marfan and Loeys-Dietz syndrome. Am. J. Med. Genet. A 161A (8), 2040–2046. 10.1002/ajmg.a.36056 23824657 PMC3885154

[B18] Schepers D. Tortora G. Morisaki H. MacCarrick G. Lindsay M. Liang D. (2018). A mutation update on the LDS-associated genes TGFB2/3 and SMAD2/3. Hum. Mutat. 39 (5), 621–634. 10.1002/humu.23407 29392890 PMC5947146

[B19] van de Laar I. M. Oldenburg R. A. Pals G. Roos-Hesselink J. W. de Graaf B. M. Verhagen J. M. (2011). Mutations in SMAD3 cause a syndromic form of aortic aneurysms and dissections with early-onset osteoarthritis. Nat. Genet. 43 (2), 121–126. 10.1038/ng.744 21217753

[B20] Vandeweyer G. Van Laer L. Loeys B. Van den Bulcke T. Kooy R. F. (2014). VariantDB: A flexible annotation and filtering portal for next generation sequencing data. Genome Med. 6 (10), 74. 10.1186/s13073-014-0074-6 25352915 PMC4210545

[B21] Ziganshin B. A. Bailey A. E. Coons C. Dykas D. Charilaou P. Tanriverdi L. H. (2015). Routine genetic testing for thoracic aortic aneurysm and dissection in a clinical setting. Ann. Thorac. Surg. 100 (5), 1604–1611. 10.1016/j.athoracsur.2015.04.106 26188975

