# Supplementary material for: Variable clinical expression of a Belgian TGFB3 founder variant suggests the presence of a genetic modifier
Source: Front Genet. 2023 Aug 31;14:1251675. doi: 10.3389/fgene.2023.1251675 (PMC10500191; doi:10.3389/fgene.2023.1251675)
Supplement: Supplementary file 1 [file Table1.DOCX]

**Supplementary Table 1:** **Overview of (likely) pathogenic *TGFB3* variants (published and current cohort).**

| **Exon/ Intron** | **c‐Notation** | **p‐Notation** | **Domain** | **Clinical Significance** | **Proband** | **Gender** | **Age last evaluation (Years)** | **Aortic manifestations** | **Non-aortic cardiovascular manifestations** | **Systemic manifestations** | **Ref.** |
| --- | --- | --- | --- | --- | --- | --- | --- | --- | --- | --- | --- |
| 1 | c.106A>T | p.Lys36* | LAP | Pathogenic | NA | M | NA | AoR aneurysm | NA | Myopia, joint dislocation, delayed wound healing | [1] |
| 1 | c.170dup | p.Glu58* | LAP | Pathogenic | Y | F | 52 | Dissection (AA, 50yrs), Ao +/- 63mm at dissection | Dilatation iliac artery at 51yrs, tortuosity (internal carotid and vertebral arteries) | Height +0.5 SD, retrognathia, myopia (−7 dioptres), DPF, crowded teeth, thin upper lip, pointed nose, kyphosis, color blindness, symptomatic hypokalemia (52yrs), varices | [2] |
|  |  |  |  |  | N | F | 50 |  | Tortuosity (internal carotid and vertebral arteries) | Hypertelorism, PE, JH, Ara, PP, EB | [2] |
| 2 | c.427A>T | p.Arg143* | LAP | Likely pathogenic | Y | M | 58 | AoR aneurysm | Trileaflet aortic valve | NA | [3] |
| 2 | c.437delT | p.Leu146Hisfs*68 | LAP | Pathogenic | NA | F | 20 | NA | NA | JH, PP, osteo-arthritis, fractures, easy bruising, food allergies | [1] |
| 2 | c.463C>T | p.(Arg155Trp) | LAP | Likely pathogenic | Y | F | 16 | NA | NA | Height +1 SD, HAP, PE, breast asymmetry, scoliosis, Ara | [2] |
| Intron 2 | c.516+1G>A | NA | NA | Likely pathogenic | Y | M | 21 | Dilatation (AoR, 16yrs) | NA | Height +3.5 DS, DC, retrognathia, myopia, DPF, HAP, cleft palate, PC, kyphoscoliosis, JH, Ara, PP, sternoclavicular subluxation, SS, autoimmune thrombocytopenia, chronic diarrhea | [2] |
| Intron 2 | c.517-3_517-2delinsAG | NA | NA | Likely pathogenic | Y | M | 50 | Dilatation (AoR, Bentall surgery 42yrs) | MVI, DCM, arrhythmia | Midface hypoplasia, HAP, scoliosis, JH, increased AHR, Ara, EB, delayed wound healing, thin translucent skin, soft velvety skin, reduced subcutaneous fat | [2] |
| 4 | c.754+2T > C | p.Glu216_Lys251del | LAP | Pathogenic | Y | F | 72 | Dissection AoA | Left subclavian artery aneurysm (45yrs) | Long face, thin skin, EB | [4] |
|  |  |  |  |  | N | M | 57 | Ruptured descending TAA (57yrs) | NA | NA | [4] |
|  |  |  |  |  | N | M | 56 | Descending TAA (50yrs), infrarenal AA (52yrs) | Carotid artery surgery | Long face | [4] |
|  |  |  |  |  | N | M | 40 | Aortic rupture (40yrs) | NA |  | [4] |
|  |  |  |  |  | N | M | 51 | Aortic sinus: 38mm | Mild MVP | PE, HAP, retrognathia, varices | [4] |
|  |  |  |  |  | N | M | 49 | Aortic sinus: 36mm | Stroke (48yrs), MI with chorda rupture surgery (49yrs) | NA | [4] |
|  |  |  |  |  | N | F | 17 | Aortic sinus: 32mm | NA | Mild scoliosis, mild JH, soft skin, EB | [4] |
|  |  |  |  |  | N | M | 24 | NA | No imaging performed | PC surgery (14yrs), HAP, Retrognathia, varices | [4] |
|  |  |  |  |  | N | F | 72 | Infrarenal AA (68yrs) | NA | Hiatal hernia | [4] |
|  |  |  |  |  | N | M | 47 | TAA | MVP with MI grade 3 | Long face, PP, hiatal hernia | [4] |
|  |  |  |  |  | N | F | 69 | Aortic sinus: 32mm, AAo: 35mm | NA | Mild scoliosis, total hip replacement, pelvic organ prolapse, hiatal hernia, varices | [4] |
|  |  |  |  |  | N | M | 44 | Aortic sinus: 39mm | Aneurysm right common iliac artery (44yrs), mild MVP | Severe scoliosis, spondylodesis Th4-L1, Long face, Retrognathia | [4] |
| 4 | c.704delA | p.Asn235Metfs*11 | LAP | Pathogenic | Y | M | 43 | TAAD type A (40yrs) | NA | Tall stature, kyphoscoliosis, pectus deformity, retrognathia, flat occiput | [4] |
|  |  |  |  |  | N | M | 80 | TAAD (80yrs) | NA | NA | [4] |
|  |  |  |  |  | N | F | 67 | TAAD type A (59yrs) | NA | Thin and tall habitus, Ara, IH, myopia | [4] |
| 4 | c.730G>A | p.(Glu244Lys) | LAP | Pathogenic | Y | F | 61 | NA | History of heart failure and hypertension, ASD and coronary artery fistulae (51yrs), spontaneous right vertebral dissection, tortuous carotid arteries | Joint laxity, atrophic scars | [5] |
| 5 | c.787G>C | p.(Asp263His) | LAP | Likely pathogenic | N | M | 49 | None | VSD, AI and MI 1/4 | IH, PP, myopia, tear ankle ligaments | [4, 6] |
|  |  |  |  |  | N | F | 37 | None | None | None | [4, 6] |
|  |  |  |  |  | Y | M | 67 | None | ICD (53yrs, polymorphic VT), concentric hypertrophy, hypertension | Peyronie | [4, 6] |
|  |  |  |  |  | N | M | 84 | None | MI 2/4, coronary artery disease hypertension | Gonarthrosis: prothesis (52yrs) | [4, 6] |
|  |  |  |  |  | N | M | 77 | None | Mild VSD (16mm), AI 2/4, mild hypertension | Shoulder tendon rupture after fall bike (76yrs) | [4, 6] |
|  |  |  |  |  | N | M | 51 | None | None | Disk herniation (35yrs), dupuytren | [4, 6] |
|  |  |  |  |  | N | M | 60 | None | None | Dupuytren | [4, 6] |
|  |  |  |  |  | N | F | 55 | None | None | EB, disk herniation, Achilles tendon rupture, rotatorcuff rupture (41yrs) | [4, 6] |
|  |  |  |  |  | N | M | 50 | NA None | VSD (14mm), mild hypertension | Myopia, PP, soft skin | [4, 6] |
|  |  |  |  |  | N | M | 7 | None | None | EB | [6] |
|  |  |  |  |  | Y | M | 65 | AA at sinus of Valsalva (50mm, z-score = 5.2), Bentall surgery | AI 3/4, MI 2/4 | Varices | [4, 6] |
|  |  |  |  |  | N | F | 55 | None | Atrial fibrillation (53yrs), hypertension | None | [4, 6] |
|  |  |  |  |  | N | F | 44 | None | None | None | [4, 6] |
|  |  |  |  |  | N | M | 81 | hypertrophy AoR aneurysm (43mm, z-score = 2.1) | pacemaker 1st grade AV block, concentric | Disk herniation | [4, 6] |
|  |  |  |  |  | N | F | 41 | None | Hypertension | None | [6] |
|  |  |  |  |  | N | M | 10 | None | None | None | [6] |
|  |  |  |  |  | Y | M | 31 | Type A aortic dissection, aortic surgery | None | Mandibular advancement surgery, long/narrow facies with mild retrognathia (post-surgery), high palate, low muscle mass, stiff joints | [4, 6] |
|  |  |  |  |  | N | M | 40 | None | None | None | [4, 6] |
|  |  |  |  |  | N | F | 71 | None | None | None | [4, 6] |
|  |  |  |  |  | N | F | 76 | None | None | None | [4, 6] |
|  |  |  |  |  | N | F | 50 | None | None | None | [4, 6] |
|  |  |  |  |  | N | M | 5 | None | None | None | [4, 6] |
|  |  |  |  |  | N | F | 14 | None | None | None | [4, 6] |
|  |  |  |  |  | Y | M | 15 | None | None | BU, tall stature, mild scoliosis, Ara, SS, PP, dolichostenomelia (armspan to height ratio: 1.05, upper segment/lower segment ratio: 0.79), positive wrist and thumb sign | [4, 6] |
|  |  |  |  |  | Y | F | 50 | None | 1^st^ grade AV block, Long QT | IH | [6] |
|  |  |  |  |  | N | M | 25 | None | None | None | [6] |
|  |  |  |  |  | N | M | 75 | AAo aneurysm (44mm, z-score=6.4) | None | Early onset varices, arthrosis | [6] |
| 5 | c.889A>G | p.(Arg297Gly) | RKKR motif | Likely pathogenic | Y | F | 10 | NA | MVP, MVI, billowing of the tricuspid valve | Height +1.5 SD, retrognathia, long face, hypertelorism, DPF, prominent eyes, HAP, scoliosis, JH, Ara, PP, camptodactyly, hammertoes, thin translucent skin, reduced subcutaneous fat, decreased muscle mass | [2] |
| 5 | c.898C > T | p.(Arg300Trp) | RKKR motif | Pathogenic | Y | M | 58 | Dilatation (AAo, AoR), David procedure (57yrs) | AI | Height +3 SD, DC, retrognathia, long face, midface hypoplasia, unilateral DPF, HAP, Ara, reduced subcutaneous fat, varices | [2] |
|  |  |  |  |  | Y | M | 6 | NA | MVP | BU, PE, JH, Ara, thoracic kyphosis, foot eversion | [2] |
|  |  |  |  |  | Y | M | 27 | Upper normal limit of AoR diameter | MVI, moderate dilatation of the left ventricle | Height +1.5 SD, exotropia, DPF, BU, HAP, PC, scoliosis, dolichostenomelia, Ara, PP, SS, dural ectasia | [2] |
|  |  |  |  |  | Y | M | 54 | Dissection (Type B extending to the AA, 52y, aortic isthmus diameter at dissection 71mm), dilatation (AoR, 52yrs) | NA | Height +1 SD, bilateral cataract (surgery 52yrs and 53yrs), myopia, BU, pneumothorax (14yrs), JH, PP, hyperelasticity of the skin, subdural hematoma, IH (53yrs), angioneurotic edema | [2] |
|  |  |  |  |  | Y | M | 14 | Dilatation (AoR, 14yrs) | NA | Height +0.5 SD, midface hypoplasia, myopia, BU, HAP, PE, increased AHR, Ara, PP | [2] |
|  |  |  |  |  | Y | F | 12 | NA | MVP | Height +0.5 SD, long face, hypertelorism, blue sclerae, BU, HAP, posterior cleft palate, JH, Ara, PP, EB, delayed wound healing, thin translucent skin, soft velvety skin | [2] |
|  |  |  |  |  | N | F | 35 | NA | NA | Height +0.5 SD, long face, smooth philtrum, hypertelorism, blue sclerae, DPF, HAP, hiatal henia (18yrs), JH, increased AHR, Ara, PP, hallux valgus, EB, delayed wound healing, thin translucent skin, soft velvety skin | [2] |
|  |  |  |  |  | N | F | 6 | NA | MVP | Height +1 SD, metopic ridge, retrognathia, long face, hypertelorism, blue sclerae, DPF, ptosis, HAP, JH, Ara, PP, hallux valgus, EB, delayed wound healing, thin translucent skin, soft velvety skin | [2] |
|  |  |  |  |  | N | M | 40 | NA | MVP, MVI | Height +2.3 SD, long face, hypertelorism, flat cornea, PC, osteoarthritis, kyphoscoliosis, JH, Ara, PP, patellar tendon rupture, fatigue, EB, thin translucent skin, soft velvety skin, skin hyperextensibility | [2] |
|  |  |  |  |  | N | M | 8 | NA | MVI (7yrs) | Height +2.5 SD, DC, long face, blue sclerae, DPF, ptosis, HAP, hypermetropia, strabismus, PE, JH, Ara, PP, genu valgum, EB, thin translucent skin, soft velvety skin, congenital hypotonia, delay of motor | [2] |
|  |  |  |  |  | Y | M | 30 | NA | Incomplete right bundle branch block | DC, BU, PE, scoliosis, JH, increased AHR, Ara, acetabular protrusion, cavus foot, SS, dural ectasia, IH | [2] |
|  |  |  |  |  | Y | F | 7 | Normal echo (4yrs) | Normal echo (4yrs) | Tall stature, cervical spine instability C2-C4, club feet, JH, hip dysplasia, BU, hypertelorism, hemiparesis, learning difficulties | [4] |
|  |  |  |  |  | N | M | 55 | NA | Cerebral aneurysm dissection | Arachnodactlyly, PE | [4] |
|  |  |  |  |  | N | F | 60 | Normal echo | Normal echo | JH, BU | [4] |
|  |  |  |  |  | N | F | 26 | Normal echo | Normal echo | AHR >1,05, BU, hypertelorysm, blue sclerae | [4] |
|  |  |  |  |  | N | M | 6 | Aortic dilatation | NA | Cleft palate, hypertelorism | [4] |
|  |  |  |  |  | N | F | 31 | Aortic sinus: 28mm (29y, z-score = -0.25) | NA | PC, cervical rib, BU, hypertelorism, translucent skin | [4] |
|  |  |  |  |  | N | M | 7 | None | Normal echo (5yrs) | Ara, JH, BU, hypertelorism | [4] |
|  |  |  |  |  | Y | M | 42 | Aortic sinus: 42mm | NA | Ara, increased AHR, retrognathia, scoliosis, PP, BU, hypertelorism, soft skin, IH surgery (6yrs) | [4] |
|  |  |  |  |  | N | F | 13 | Normal echo | Normal echo | Ara, asymmetric chest, narrow palate with dental crowding, cleft palate, IH | [4] |
|  |  |  |  |  | N | F | 10 | Normal echo | Normal echo | Cleft palate | [4] |
|  |  |  |  |  | Y | M | 24 | Aortic sinus: 40mm (z-score = 3.6) | MVP, PFO | PC, PP, BU, cleft palate, hypertelorism, club foot | [4] |
|  |  |  |  |  | Y | F | 3 | AoR aneurysm: 19.5mm (Z>2) | VSD+ASD | NA | [4] |
|  |  |  |  |  | N | M | 27 | NA | NA | Tall stature, Ara, JH, HAP, kyphosis, PP, hypertelorism, BU, spondylolisthesis | [4] |
| 5 | c.899G > A | p.(Arg300Gln) | RKKR motif | Pathogenic | Y | M | 44 | NA | NA | Height +3 SD, PC, JH, increased AHR, clubfeet, coxarthrosis, varices | [7] |
|  |  |  |  |  | N | M | 4 | NA | MVP | Height +2 SD, DC, long face, hypertelorism, myopia, BU, high forehead, frontal bossing, short philtrum, PE, JH, PP, congenital hypotonia (mild), delay of motor development | [7] |
|  |  |  |  |  | N | F | 52 | NA | NA | Height +3 SD, long face, prominent eyes, PC, bilateral coxa valga, JH, increased AHR, Ara, left PP, right pes cavus, clubfeet, EB, soft velvety skin, decreased muscle mass | [7] |
|  |  |  |  |  | N | M | 8 | NA | NA | Height +2 SD, HAP, anomaly of teeth position, PP, unilateral IH | [7] |
|  |  |  |  |  | Y | F | NA | NA | Arterial tortuosity | BU, JH, PP, pectus deformity, scoliosis, osteo-arthritis, PE | [1] |
| 5 | c. 898C > G | p.(Arg300Gly) | RKKR motif | Likely pathogenic | N | F | 13 | NA | NA | Slender build, reduced subcutaneous fat, low muscle mass, muscular hypotonia, tall stature, long and slender fingers/toes, PP, JH, positive wrist signs, mild scoliosis, hypertelorism, small chin, crowded teeth, BU | [8] |
|  |  |  |  |  | N | M | 9 | NA | NA | Slender build, reduced subcutaneous fat, low muscle mass, muscular hypotonia, tall stature, long and slender fingers/toes, PP, JH, positive wrist signs, mild scoliosis, hypertelorism, small chin, crowded teeth, BU, bilateral IHs | [8] |
|  |  |  |  |  | N | M | 41 | Normal echocardiography | Arterial hypertension, normal echocardiography | Tall stature (196cm +2,7SD), used to be slender, long hands/fingers and feet/toes, IHs as a child, hypertelorism, small chin, BU | [8] |
| 5 | c.908A>C | p.(Asp303Ala) | Cytokine | VUS^a^ | Y | M | 52 | Dilatation (AoR, 52yrs) | MVP, MVI | Height −0.5 SD, PE, osteoarthritis, increased AHR, osteoporosis (51yrs), kyphosis, pinching vertebral discs, reduced subcutaneous fat | [2] |
| 6 | c.952C>T | p.(Arg318Cys) | Cytokine | Likely pathogenic | Y | M | 18 | NA | NA | Height +2 SD, DC, BU, HAP, cleft palate, PE, spondylolisthesis, JH, Ara, PP, decreased muscle mass | [2] |
| 6 | c.965T > C | p.(Ile322Thr) | Cytokine | Likely pathogenic | Y | M | 56 | AAA with Y-graft (43yrs), TAA (47mm) with severe AR: Bentall surgery | NA | Tall stature, Ara, kyphoscoliosis, pectus deformity, retrognathia, down-slanting palp fissures, hypertelorism, osteoarthritis | [4] |
|  |  |  |  |  | N | M | 24 | AoR 32mm | NA | Tall stature, Ara, scoliosis, SS | [4] |
|  |  |  |  |  | N | F | 22 | AoR 34mm | NA | Tall stature Ara, JH, kyphoscoliosis, PP, retrognathia, down-slanting palp fissures, hypertelorism, BU | [4] |
| 6 | c.979G>T | p.(Asp327Tyr) | Cytokine | Likely pathogenic | Y | M | 26 | AoR aneurysm | NA | Hypertelorism, DPS, dolichostenomelia, JH, Ara, SS | [1] |
|  |  |  |  |  | N | M | NA | AoR aneurysm | NA | Hypertelorism, BU |  |
|  |  |  |  |  | N | F | NA | Aortic tortuosity | NA | DPS, scoliosis, delayed wound healing, PC |  |
| 6 | c.1075A>C | p.(Ser359Arg) | Cytokine | VUS^a^ | Y | M | 60 | Dilatation (AoR46mm, 25yrs; AoR 55mm, 57yrs; AAo 48mm, 57yrs) | AI (25yrs), PSVT (55yrs) | Height 0 SD, myopia, amblyopia, DPF, HAP, PP (during childhood), UH | [9] |
|  |  |  |  |  | N | F | 33 | NA | NA | Height −1 SD, hypermetropia | [9] |
|  |  |  |  |  | N | F | 58 | NA | NA | Height −0.6 SD, unilateral cataract (surgery at 49yrs), myopia | [9] |
| Intron 6 | c.1081-2A>T | NA | NA | Pathogenic | Y | F | 22 | Dilatation (AoR, 17yrs) | NA | Height +1.5 SD (target height −0.3 SD) retrognathia, long face, hypertelorism, myopia, DPF, prominent eyes, low-set ears, overbite, BU, cleft palate, PE, spondylolisthesis, scoliosis, increased AHR, Ara, PP, thin translucent skin, reduced subcutaneous fat, decreased muscle mass, diaphragmatic hernia, UH | [2] |
|  |  |  |  |  | N | M | 18 | NA | NA | Height −1 SD, BU, dental crowding, PP, congenital hip dysplasia | [2] |
|  |  |  |  |  | N | F | 10 | NA | NA | Height +1 SD, myopia (−5.5/−5.75 dioptres), HAP | [2] |
|  |  |  |  |  | N | F | 41 | NA | NA | Height 0 SD, 163.5cm, DPF, HAP, broad uvula | [2] |
|  |  |  |  |  | N | M | 49 | Dilatation (AoR 39mm, 49yrs) | NA | Height −0.5 SD, hypertelorism, bilateral cataract (47yrs), myopia (−16 dioptres), broad uvula, mild PC, increased AHR, osteoporosis, EB, thin translucent skin, unilateral IH (34yrs), UH, varices | [2] |
| 7 | c.1095C > A | p.Tyr365* | Cytokine | Pathogenic | Y | F | 42 | Normal echo | Normal echo | Ara, JH, camptodactyly toes, PP, BU, hypertelorism, EB, delayed motor development, varices, cataract | [4] |
| 7 | c.1044C>A | p.Cys384* | Cytokine | Pathogenic | Y | M | 54 | Stanford type A, Debakey type I aortic dissection in 2018. Follow up CT angiography: rapid dilatation of proximal desc Ao. 2019: AoA replacement. | CCA dissection, occluded left CCA. Asymptomatic small-sized acute ischemic infarct in right superior frontal gyrus. | Joint dislocations, skin hyperextensibility, JH, PC, bilateral pes cavus, BU, UH | [10] |
| 7 | c.1157delT | p.Leu386Argfs*21 | Cytokine | Pathogenic | Y | M | 43 | NA | MVP | DC, tall stature, Ara, scoliosis, PP, BU, hypertelorysm | [4] |
|  |  |  |  |  | N | M | 70 | TAA (61yrs) with TAAD (70yrs) | NA | Tall stature, cleft palate, BU | [4] |
|  |  |  |  |  | N | F | NA | NA | NA | Tall stature, Ara | [4] |
|  |  |  |  |  | N | F | 14 | Normal echocardiography | Normal echocardiography | Tall stature, HAP, Ara, scoliosis | [4] |
| 7 | c.1202T > C | p.(Leu401Pro) | Cytokine | Likely pathogenic | Y | M | 50 | AAo 60mm (42yrs), type A dissection with supracoronary replacement (43yrs), TEVAR for type B dissection (44yrs), AAA (50yrs) | NA | Increased AHR, Ara, DC, mild scoliosis, hypertelorism, spondylosis C3-C4, C5-C6 and C6-C7, bilateral IH surgery (39yrs) | [4] |
|  |  |  |  |  | N | F | 30 | Normal echocardiography | Normal echocardiography | Increased AHR, Ara, scoliosis, JH, metatarsus adductus, camptodactyly of 4th/5th toes | [4] |
| 7 | c.1226G > A | p.(Cys409Tyr) | Cytokine | Pathogenic | Y | F | 9 | NA | NA | Contractures in hands and toes, long and narrow hands and feet (no measurements), midline facial nevus flammeus, mild hypotonia, failure to thrive because of low weight (3mo), delay in gross motor function, small and tall, bilateral PP, mild PE, hyperextensibililty, mild retrognathia, prominent eyes, hypertelorism, BU, decreased bulk in all axial muscles, reduced strength, low tone, diminished reflexes, reduced subcutaneous fat | [11] |
| *^a^ Variant is included because of its location within the TGFß3 cytokine domain which gives a stronger indication to be likely pathogenic and the presence of clinical information.* | | | | | | | | | | | |
| *AA, aortic aneurysm; AAA, abdominal aortic aneurysm; AAo, ascending aorta; AHR, arm/height ratio; AI, aortic insufficiency; AoA, aortic arch; AoR, aortic root; Ara, arachnodactyly; ASD, atrial septal defect; AV, Atrioventricular; BU, bifid uvula; CCA, common carotid artery; CT, computed tomography; DC, dolichocephaly; DCM, dilated cardiomyopathy; DPF, downslanting palpebral fissures; EB, easy bruising; HAP, highly arched palate; ICD, implantable cardioverter-defibrillator; IH, inguinal hernia; JH, joint hypermobility; LAP, latency-associated peptide; MI, myocardial infarction; MVI, mitral valve insufficiency; MVP, mitral valve prolapse; NA, not applicable/no information available; PC, pectus carinatum; PE, pectus excavatum; PFO, patent foramen ovale; PP, pes plani; PSVT, paroxysmal supraventricular tachycardia; SS, skin striae; TAA, thoracic aortic aneurysm; TEVAR, thoracic endovascular aortic repair; UH, umbilical hernia; VSD, ventricular septal defect; VT, ventricular tachycardia; VUS, variant of uncertain significance.* | | | | | | | | | | | |

# Supplementary References

1. Schepers, D., et al., *A mutation update on the LDS-associated genes TGFB2/3 and SMAD2/3.* Hum Mutat, 2018. **39**(5): p. 621-634.

2. Marsili, L., et al., *Phenotypic spectrum of TGFB3 disease-causing variants in a Dutch-French cohort and first report of a homozygous patient.* Clin Genet, 2020. **97**(5): p. 723-730.

3. Ziganshin, B.A., et al., *Routine Genetic Testing for Thoracic Aortic Aneurysm and Dissection in a Clinical Setting.* Ann Thorac Surg, 2015. **100**(5): p. 1604-11.

4. Bertoli-Avella, A.M., et al., *Mutations in a TGF-beta ligand, TGFB3, cause syndromic aortic aneurysms and dissections.* J Am Coll Cardiol, 2015. **65**(13): p. 1324-1336.

5. Abdelhadi, N., et al., *Recurrent Coronary Artery Fistulae and a Novel Transforming Growth Factor Beta-3 Mutation.* Cureus, 2021. **13**(9): p. e17780.

6. Current study.

7. Matyas, G., et al., *De novo mutation of the latency-associated peptide domain of TGFB3 in a patient with overgrowth and Loeys-Dietz syndrome features.* Am J Med Genet A, 2014. **164A**(8): p. 2141-3.

8. Kuechler, A., et al., *Exome sequencing identifies a novel heterozygous TGFB3 mutation in a disorder overlapping with Marfan and Loeys-Dietz syndrome.* Mol Cell Probes, 2015. **29**(5): p. 330-4.

9. Overwater, E., et al., *Results of next-generation sequencing gene panel diagnostics including copy-number variation analysis in 810 patients suspected of heritable thoracic aortic disorders.* Hum Mutat, 2018. **39**(9): p. 1173-1192.

10. Hussein, D., et al., *Novel Mutation of the TGF-beta 3 Protein (Loeys-Dietz Type 5) Associated With Aortic and Carotid Dissections: Case Report.* Neurol Genet, 2021. **7**(6): p. e625.

11. Rienhoff, H.Y., Jr., et al., *A mutation in TGFB3 associated with a syndrome of low muscle mass, growth retardation, distal arthrogryposis and clinical features overlapping with Marfan and Loeys-Dietz syndrome.* Am J Med Genet A, 2013. **161A**(8): p. 2040-6.
