# Supplementary material for: Variable clinical expression of a Belgian TGFB3 founder variant suggests the presence of a genetic modifier
Source: Front Genet. 2023 Aug 31;14:1251675. doi: 10.3389/fgene.2023.1251675 (PMC10500191; doi:10.3389/fgene.2023.1251675)
Supplement: Supplementary file 2 [file Table2.DOCX]

**Supplementary Table 2:** Composition of the Haloplex based TAAD_v9 gene panel.

| **Gene** | **Transcript reference**  **(Ensembl)** | **Alternative exon** | **Transcript reference for alternative exon (Ensembl)** |
| --- | --- | --- | --- |
| *ABL1* | ENST00000372348 |  |  |
| *ACTA2* | ENST00000458208 |  |  |
| *ARIH1* | ENST00000379887 |  |  |
| *BGN* | ENST00000331595 |  |  |
| *COL3A1* | ENST00000304636 |  |  |
| *EFEMP2/FBLN4* | ENST00000307998 |  |  |
| *ELN* | ENST00000358929 |  |  |
| *EMILIN1* | ENST00000380320 |  |  |
| *FBN1* | ENST00000316623 |  |  |
| *FBN2* | ENST00000262464 |  |  |
| *FLNA* | ENST00000369850 |  |  |
| *FOXE3^a^* | ENST00000335071 |  |  |
| *HCN4* | ENST00000261917 |  |  |
| *LMOD1* | ENST00000367288 |  |  |
| *LOX* | ENST00000231004 |  |  |
| *LTBP3* | ENST00000301873 |  |  |
| *MAT2A* | ENST00000306434 |  |  |
| *MFAP5* | ENST00000359478 |  |  |
| *MYH11* | ENST00000452625 | Exon 42B | ENST00000396324 |
| *MYLK* | ENST00000360304 |  |  |
| *NOTCH1* | ENST00000277541 |  |  |
| *PLOD1* | ENST00000196061 | Exon 2A | ENST00000449038 |
| *PMEPA1/TMEPAI* | ENST00000341744 |  |  |
| *PRKG1^b^* | ENST00000401604 |  |  |
| *SKI* | ENST00000378536 |  |  |
| *SLC2A10* | ENST00000359271 |  |  |
| *SMAD2* | ENST00000402690 |  |  |
| *SMAD3* | ENST00000327367 | Exon 1A | ENST00000439724 |
| *SMAD4* | ENST00000342988 |  |  |
| *SMAD6* | ENST00000288840 |  |  |
| *TGFB2* | ENST00000366929 |  |  |
| *TGFB3* | ENST00000238682 |  |  |
| *TGFBR1^c^* | ENST00000374994 |  |  |
| *TGFBR2* | ENST00000359013 |  |  |
| *^a^ Only Forkhead domain*  *^b^ Only exon3* | |  |  |
